# Polyethylenimine-Functionalized Nanofiber Nonwovens Electrospun from Cotton Cellulose for Wound Dressing with High Drug Loading and Sustained Release Properties

**DOI:** 10.3390/polym14091748

**Published:** 2022-04-26

**Authors:** Qunhao Wang, Mei Li, Zhuo Zheng, Yan Niu, Xiaolin Xue, Chenghong Ao, Wei Zhang, Canhui Lu

**Affiliations:** 1 State Key Laboratory of Polymer Materials Engineering, Polymer Research Institute, Sichuan University, Chengdu 610065, China; wangqunhao@stu.scu.edu.cn (Q.W.); limei991010@163.com (M.L.); zhuo_zheng@scu.edu.cn (Z.Z.); 17839918692@163.com (Y.N.); xuexiaolin@stu.scu.edu.cn (X.X.); chenghongaocd@163.com (C.A.); 2 Faculty of Environmental Science & Engineering, Kunming University of Science & Technology, Kunming 650500, China; 3 Advanced Polymer Materials Research Center, Sichuan University, Shishi 362700, China

**Keywords:** cellulose, drug carrier, electrospinning, polyethylenimine, wound dressing

## Abstract

Electrospun cellulose nanofiber nonwovens have shown promise in wound dressing owing to the highly interconnected pore structure, high hydrophilicity coupled with other coveted characteristics of biodegradability, biocompatibility and renewability. However, electrospun cellulose wound dressings with loaded drugs for better wound healing have been rarely reported. In this study, a novel wound dressing with a high drug loading capacity and sustained drug release properties was successfully fabricated via electropinning of cellulose followed by polyethylenimine (PEI)-functionalization. Remarkably, the grafted PEI chains on the surface of electrospun cellulose nanofibers provided numerous active amino groups, while the highly porous structure of nonwovens could be well retained after modification, which resulted in enhanced adsorption performance against the anionic drug of sodium salicylate (NaSA). More specifically, when immersed in 100 mg/L NaSA solution for 24 h, the as-prepared cellulose-PEI nonwoven displayed a multilayer adsorption behavior. And at the optimal pH of 3, a high drug loading capacity of 78 mg/g could be achieved, which was 20 times higher than that of pristine electrospun cellulose nonwoven. Furthermore, it was discovered that the NaSA-loaded cellulose-PEI could continuously release the drug for 12 h in simulated body fluid (SBF), indicating the versatility of cellulose-PEI as an advanced wound dressing with drug carrier functionalities.

## 1. Introduction

Electrospinning is a simple and effective fiber processing technique, which employs high voltage electric field to produce ultrathin fibers with diameters down to the nano range from a broad spectrum of polymers [1,2]. Electrospun nanofibers present many unique characteristics, such as high porosity, high aspect ratio and large specific surface area, demonstrating great potentials in various end-uses, such as environmental remediation, energy storage, catalysis and biomedicine, just name a few [3,4,5]. In particular, the electrospun nanofiber nonwovens can well mimic the natural extracellular matrix (ECM) structure and thus promote cell migration and proliferation [6]. Moreover, the small pores inside the nonwovens can effectively prevent bacterial invasion, while their high surface area favors fluid absorption and dermal drug delivery. Therefore, the electrospun nanofiber nonwovens are regarded as an ideal material for wound dressing. So far, a variety of electrospun nanofibers from synthetic polymers, such as polyurethane and poly(vinyl pyrrolidone)-iodine, have been fabricated and applied in wound dressings [7]. However, due to the intrinsic drawbacks of raw materials (nonrenewability, poor wettability, inferior biocompatibility and biodegradability), their wound dressing performance is still far from expectation. 

In recent years, the global issues caused by synthetic polymers, namely, environmental pollution and shortage of chemical feedstocks (e.g., crude oil), have triggered intense research interests in renewable resources [8,9]. Cellulose is one of the most abundant natural polymers on earth with many outstanding properties [10,11,12,13,14]. Of particular note, cellulose based materials (e.g., cotton bandage) have long been practiced in wound dressing for the excellent biocompatibility and high hydrophilicity [15]. However, these conventional cellulose wound dressings have some obvious limitations. The pore size is always too large to inhibit bacteria penetration. Additionally, their coarse surface texture also brings about uncomfortable feelings to the wound site especially when in movement. To address these issues, the electrospinning technology has been applied to prepare nonwoven wound dressings. Comprised of randomly packed nanofibers, the electrospun cellulose nonwovens exhibit some extraordinary attributes. They have numerous interconnected pores with tunable sizes, highly accessible and reactive surface functional groups, superior mechanical property as well as structural similarity to natural ECM [16], making them a promising candidate for wound care [17]. Previously, a flexible nanocomposite nonwoven based on cellulose and gelatin was electrospun and applied as a wound dressing material [18]. This nonwoven membrane showed excellent mechanical strength, water vapor permeability, water-uptake capacity and biocompatibility, which promoted wound healing. In another study, an antibacterial chitosan/bacterial cellulose nonwoven was produced by parallel and coaxial electrospinning [19]. It exhibited superb biocompatibility, regenerative property, water holding capacity and mechanical property, which were desired for wound dressing applications. 

Drug-loaded wound dressing offers the prospect of delivering the drug directly to the target site. As such, some detrimental side effects can be effectively prohibited in comparison with oral or parenteral administration protocols [20]. Although cellulose has a plenty of hydroxyls on its molecular chains which may establish hydrogen bonding interactions with drugs, its drug loading capacity is usually too low to provide an effective therapy. To overcome this problem, several chemical modification methods have been disclosed, which resulted in remarkably improved drug loading capacity of cellulose [21,22]. For those drugs with negatively charged ions, amino functionalization of cellulose is a common strategy to strengthen electrostatic interaction with the drugs [23].

Herein, we report a novel wound dressing material with sustained drug release profiles by first electrospinning of the cellulose solution into nanofiber nonwovens followed by surface chemical modification with polyethylenimine (PEI). Note that PEI contains abundant primary and second amino groups and it is an ordinary cross-linking agent as well as a surface functional modifier for cellulose [23,24,25]. In this study, PEI offered numerous active amino groups to bind more drug molecules. An anti-inflammatory anionic drug of sodium salicylate (NaSA) capable of promoting wound healing and treating diabetes [26], arthritis [27], and cancers [28], was used as the model drug. The effects of pH, drug concentration and contact time on the drug loading capacity of cellulose-PEI were studied. And the Langmuir and Freundlich models were employed to investigate the drug adsorption behavior. Furthermore, the in vitro drug release behavior of the NaSA-loaded cellulose-PEI membrane was analyzed in simulated body fluid (SBF).

## 2. Materials and Methods

### 2.1. Materials

Medical level cotton was purchased from Health Materials Co., Ltd., Chengdu, China. Methanol, Sodium salicylate, Ethylenediamine, Glutaraldehyde, Acetone, Cerium ammonium nitrate, Nitric acid, Methyl methacrylate, N, N-dimethylacetamide (DMAc) and Lithium chloride (LiCl) were all supplied by Kelong Chemicals Co., Ltd., Chengdu, China. Polyethylenimine (MW of 25 kg/mol) was purchased from Sigma-Aldrich, St. Louis, MO, USA.

### 2.2. Preparation of Cellulose Solution

The protocol for cellulose dissolution followed the method in literature [7]. Briefly, the cotton was firstly activated by sequential immersion in water, methanol and DMAc for 1 h, respectively, followed by vacuum filtration. A DMAc/LiCl solution with a mass ratio of 92:8 was prepared as the solvent for cellulose [29]. Then, 1 g activated cotton was added into 99 g DMAc/LiCl solvent and continuously stirred at room temperature for 24 h to obtain an optically transparent cellulose solution. The whole preparation process was maintained in air-free conditions to avoid moisture absorption.

### 2.3. Preparation of Electrospun Cellulose Nonwoven

The electrospinning process was in accordance with our previous work [29]. Briefly, the cellulose solution was transferred into a 20 mL plastic syringe with a 0.8 mm stainless steel needle. Then, the syringe was loaded onto an electrospinning equipment (FM-12, Fuyouma Technology Co., Ltd., Tianjin, China) with a micro pump to control the flow rate (1.8 mL/h) of the solution. The needle was connected to a high voltage power supply, which was set to generate a positive voltage up to 20 kV. A steel rotating collector (6 cm in diameter) wrapped with an aluminum foil was placed 10 cm away from the nozzle tip and the rotate speed was fixed at 120 r/min to help collect electrospun fibers. Importantly, the rotating collector was partially immersed in a water coagulation bath (about 1 cm in depth) for thorough removal of residual solvent so that dimensionally stable electrospun nanofiber nonwovens could be produced.

### 2.4. Preparation of Cellulose-NH_2_ Membrane

1 g of as-prepared electrospun cellulose nonwoven was immersed in 10 mL methanol under nitrogen atmosphere. Then, 1.5 g methyl methacrylate (MMA) and 6 mM of cerium ammonium nitrate were slowly added into the aforementioned solution for polymerization. The reaction lasted for about 3 h under continuous stirring at room temperature. After that, the product was washed with acetone and methanol for several times. Next, the product and 1.5 g ethylenediamine were added into methanol and stirred at 60 °C for 24 h. After washing with methanol for three times, the cellulose-NH_2_ membrane was ultimately obtained.

### 2.5. Preparation of Cellulose-PEI Membrane 

2 g cellulose-NH_2_ was soaked into 100 mL PEI solution in methanol (2 wt%) and stirred for 24 h at room temperature. After that, 100 mL glutaraldehyde solution in methanol (1 wt%) was added into the mixture and further stirred for 30 min to obtain the cellulose-PEI membrane. The residual methanol in the membrane was removed by washing with distilled water for several times. And the membrane was finally subjected to freeze-drying (FD-1A-50, Yaxing Yike Technology Co., Ltd., Beijing, China) for 48 h.

### 2.6. Material Characterization

The morphologies of cellulose-PEI membrane and pristine electrospun cellulose were observed using a scanning electron microscopy (SEM, Inspect F 50, FEI, Waltham, Massachusetts, United States) at 20 kV. The chemical structure of products was characterized by Fourier transform infrared spectroscopy (FTIR) and X-ray Photoelectron Spectroscopy (XPS), respectively. FTIR analysis was performed using a FTIR spectrometer (Nicolet 560, Nicolet, Madison, WI, USA). The spectra were scanned from 4000 to 500 cm^−1^ with a resolution of 2 cm^−1^. The XPS spectra were recorded on an XPS spectrometer (XASAM 800, Kratos Analysis, Manchester, UK) with an Al Kα X-ray source (1486.6 eV) and an X-ray beam of around 1 mm. The concentration of NaSA was measured at 295 nm on a UV−vis spectrophotometer (UV-1600, Mapada, Shanghai, China). 

### 2.7. Standard Curve Measurement

The light absorbance of NaSA solutions with different concentrations (0.5 mg/L, 1.0 mg/L, 2.0 mg/L, 10.0 mg/L, 20.0 mg/L, 50.0 mg/L, 100.0 mg/L) at 295 nm was measured on the UV-vis spectrophotometer to obtain the standard absorbance-concentration curve of NaSA solutions.

### 2.8. Drug Adsorption

The cellulose-PEI and neat electrospun cellulose were loaded with NaSA via adsorption. Firstly, the analytical grade NaSA was dissolved into twice-distilled water and then diluted into different target concentrations. In the subsequent adsorption process, the neat electrospun cellulose and cellulose-PEI were added into the NaSA solution, respectively, and shaken at 25 °C for 24 h for sufficient drug adsorption. The drug loading of materials could be determined from the initial and final drug concentrations of solutions from the UV-vis spectrophotometer and calculated following the Equation (1).

(1) $$q_{e}=\frac{\left(C_{0}-C_{e}\right)\times V}{m}$$

where C_0_ and C_e_ (mg/L) are the initial and equilibrium concentrations of NaSA, respectively. V (L) is the volume of drug solution, m (g) is the mass of sorbents, q_e_ (mg/g) is the equilibrium adsorption capacity of sorbents.

### 2.9. Drug Release Study

Considering the fact that the ambient human body condition is constant (37 °C, pH = 7.4), the simulated body fluid (SBF) was chosen as the medium for drug release study. The cellulose-PEI membrane was first loaded with drug in the 100 mg/L NaSA solution for 24 h (25 °C, pH = 3) and then freeze-dried at −40 °C for 48 h. After that, the material was soaked into SBF (pH = 7.4) and shaken at 37 °C. The concentration of drug in the SBF was measured at preset intervals to investigate the drug release performance.

## 3. Results and Discussion

### 3.1. Fabrication of Cellulose-PEI Nonwoven Membranes

**Figure 1:**
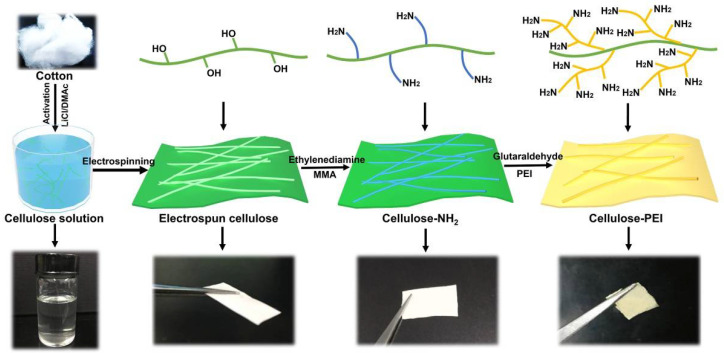
Schematic for the preparation process of cellulose-PEI.

The essence of our strategy to prepare cellulose-PEI was schematically illustrated in Figure 1. First, the cellulose nonwoven membrane was fabricated via electrospinning of a cellulose solution prepared by dissolving cotton in the DMAc/LiCl solution. Second, MMA polymerized on the surface of cellulose nanofibers and then reacted with ethylenediamine to introduce amino groups. Third, the cellulose-NH_2_ membrane was soaked in a PEI solution in methanol and a glutaraldehyde solution in methanol was then added for further reaction. Finally, PEI was successfully grafted onto cellulose-NH_2_ to obtain cellulose-PEI with abundant amino groups.

### 3.2. Morphological and Structural Characterization of Cellulose-PEI

**Figure 2:**
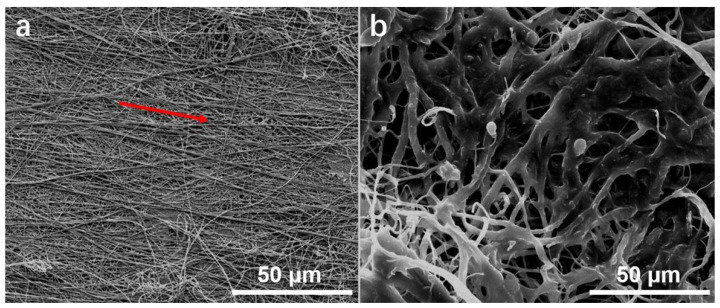
The SEM images of neat electrospun cellulose (**a**) and cellulose-PEI (**b**).

The morphologies of neat electrospun cellulose and cellulose-PEI were observed on SEM, and the representative images were shown in Figure 2. The neat electrospun fibers displayed uniform diameter distribution with obvious alignment tendency (indicated with a red arrow), which was attributed to the rotating collector used during electrospinning. After PEI modification, the fibers apparently became much thicker (Figure 2b), owing to the fact that some of the initial nanofibers were bundled and wrapped by PEI during the surface modification process. It is noteworthy that cellulose-PEI well retained the highly porous structure of its precursor nonwoven, which can facilitate the flow of drugs into its interior. With the presence of PEI on the fiber surface, cellulose-PEI should adsorb cationic drugs in the solution more efficiently [30].

**Figure 3:**
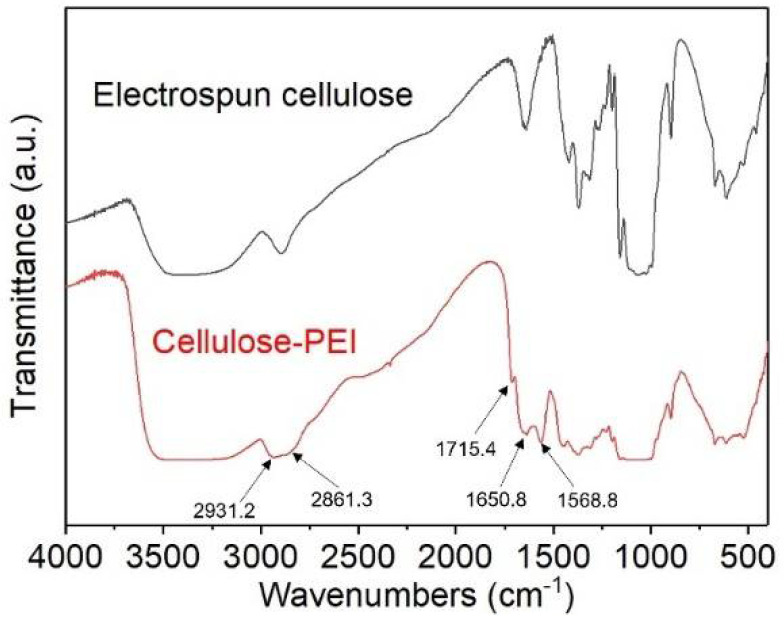
The FTIR spectra of neat electrospun cellulose and cellulose-PEI.

The chemical structures of the electrospun cellulose before and after PEI grafting were verified by FTIR analysis (Figure 3). Compared with neat electrospun cellulose, cellulose-PEI displayed distinct absorption bands at 2931.2, 2861.3, 1715.4, 1650.8 and 1568.8 cm^−1^ on its FTIR spectrum. The characteristic band at around 1715.4 cm^−1^ should be assigned to the C=O stretching of the ester groups in methyl methacrylate [31], indicating that methyl methacrylate had been grafted onto the electrospun cellulose. Meanwhile, it also suggested that the reaction between the ester groups and ethylenediamine was not complete [31]. The peaks at 1650.8 cm^−1^ corresponded to the C=N stretching vibration, which was formed in the glutaraldehyde crosslinking process [32]. The peak at 1568.8 cm^−1^ was assigned to the N−H bending vibration of the primary amines in amino groups [33,34], which confirmed the successful grafting of PEI on cellulose. Furthermore, the occurrence of −CH_2_ stretching vibrations at 2931.2 cm^−1^ and 2861.3 cm^−1^ for cellulose-PEI also proved the successful grafting of PEI onto the electrospun cellulose [35]. 

**Figure 4:**
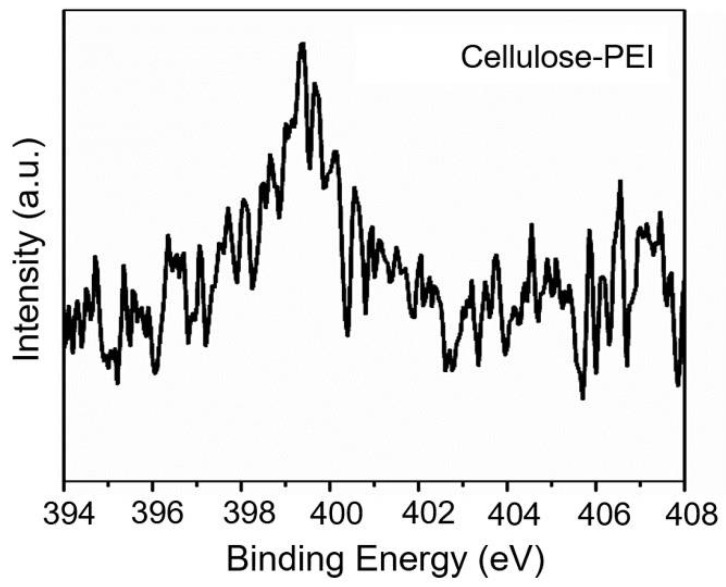
XPS spectrum of cellulose-PEI.

XPS is a powerful tool to analyze the chemical composition of materials [36]. As shown in Figure 4, the cellulose-PEI exhibited an evident peak of N_1s_ at 399.3 eV, and the content of nitrogen was calculated to be 3.95%, which consistently manifested that PEI had been grafted onto electrospun cellulose [33]. Note that these accessible amino groups could provide abundant binding sites for anionic drugs, thus greatly enhancing the drug loading capacity of cellulose-PEI [37]. 

### 3.3. Drug Adsorption Studies

NaSA was selected as a model drug to investigate the drug adsorption performance of cellulose-PEI. From the UV-vis spectra, a standard absorbance-concentration curve for NaSA solutions was plotted with linear fitting (Figure S1). And the linear fitting parameter R^2^ was very high (0.99984).

#### 3.3.1. The Effect of pH on the Drug Adsorption Capacity

**Figure 5:**
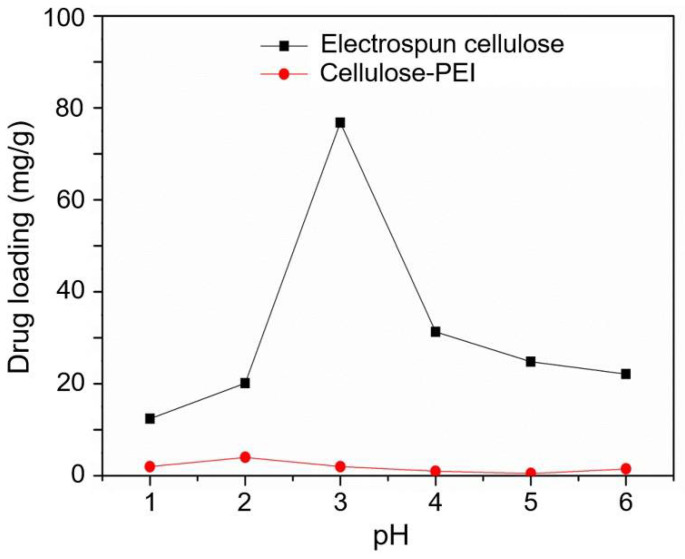
Drug adsorption capacities of the neat electrospun cellulose and cellulose-PEI at different pH values.

To investigate the effect of pH on the adsorption capacity, both of electrospun cellulose and cellulose-PEI were immersed in the NaSA solution (100 mg/L) for 24 h at various pH values (1 to 6). As shown in Figure 5, the NaSA loading capacity of cellulose-PEI was much higher than that of neat electrospun cellulose in the entire pH range, indicating that PEI modification could significantly improve the drug loading capacity of the electrospun cellulose nonwoven. In detail, the adsorption capacity of cellulose-PEI for NaSA increased with the increase of pH from 1 to 3. At pH = 3, its maximum adsorption capacity reached 78 mg/g, which was 20 times higher than that of neat electrospun cellulose. However, the drug adsorption would decrease when pH was further increased from 3 to 6. Such adsorption behaviors should be presumably ascribed to the changes of interaction between the surface charges of cellulose-PEI and SA^−^ at different pH values. Under acid conditions, the amino groups would be protonated to form positively charged active sites, such as −NH_3_^+^ [24]. The electrostatic interaction between −NH_3_^+^ and SA^−^ was essential for the improvement of adsorption property. The bell-shaped curve could be predicted based on pKa values with the Henderson−Hasselbalch equation [38].

(2) $$\mathrm{pH}={\mathrm{pk}}_{a1}+\log\frac{\left[{\mathrm{SA}}^{-}\right]}{\left[\mathrm{SA}\right]}$$

(3) $$\mathrm{pH}={\mathrm{pK}}_{a2}+\log\frac{\left[{\mathrm{NH}}_{2}\right]}{\left[{\mathrm{NH}}_{3}{}^{+}\right]},$$

According to Equations (2) and (3), with the increase of pH, the amino groups could hardly be protonated and the concentration of −NH_3_^+^ in the fluid decreased, but the concentration of SA^−^ would increase. At pH of 1–3, the concentration of SA^−^ was at a relatively low level (pK_a1_ = 3) [39]. Therefore, the NaSA loading mainly depended on the concentration of SA^−^, resulting in drug adsorption capacity increase with the increase of pH. At pH of 3, the amino groups on cellulose-PEI (pK_a2_ = 8) [40] were extensively protonated to bare positive charges. Simultaneously, a half of SA was presented in the form of SA^−^. For the strong electrostatic attraction between SA^−^ and −NH_3_^+^, the drug loading reached the maximum at pH of 3. However, when pH was further increased (pH > 3), the concentration of −NH_3_^+^ would decrease. In this situation, the NaSA loading should be mainly governed by the concentration of NH_3_^+^, leading to the decay of drug adsorption capacity with the increase of pH.

#### 3.3.2. The Effect of Drug Concentration on Adsorption Capacity

**Figure 6:**
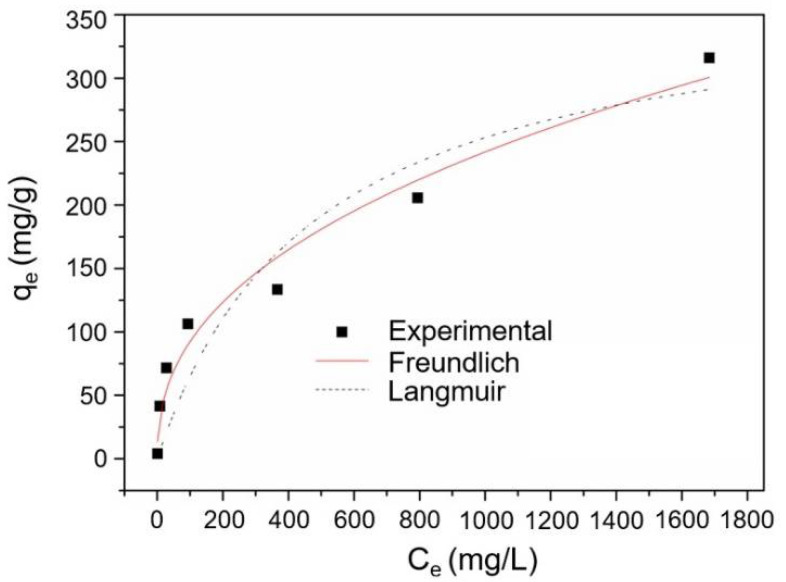
The drug adsorption isotherms for cellulose-PEI.

The drug adsorption capacities of cellulose-PEI were investigated under different NaSA concentrations at pH of 3. As shown in Figure 6, the adsorption capacity of cellulose-PEI increased with the increase of NaSA concentration. Nobly, the equilibrium drug adsorption capacity (q_e_) of cellulose-PEI exceeded 300 mg/g when the equilibrium drug concentration of the solution (C_e_) was around 1.7 g/L. A high drug loading capacity offered cellulose-PEI with tremendous potentials in future drug carrier design.

To better understand the interaction between NaSA and cellulose-PEI, the adsorption isotherms of cellulose-PEI were fitted by Langmuir model [41] (Equation (4)) and Freundlich [25] (Equation (5)) model.

(4) $${\;q}_{e}=\frac{q_{m}K_{L}C_{e}}{1+K_{L}{\mathrm{XC}}_{e}}$$

(5) $${\;q}_{e}={\;K}_{F}C_{e}{}^{\frac{1}{n}}$$

where C_e_ (mg/L) is the equilibrium concentration of NaSA, q_e_ (mg/g) is the equilibrium adsorption capacity of adsorbents, q_m_ (mg/g) is the maximum adsorption capacity of adsorbents, K_L_ and K_F_ are constants for Langmuir and Freundlich models, respectively, n is a Freundlich constant relating to adsorption intensity of the adsorbents.

The fitting curves and the corresponding fitting parameters were shown in Figure 6 and Table 1, respectively. The regression coefficient (R^2^) of Freundlich model was 0.9681, much higher than that of Langmuir model, indicating that the Freundlich isotherm could better describe the drug adsorption behavior of cellulose-PEI and it was a multilayer adsorption process [25]. 

### 3.4. Drug Release Study

**Figure 7:**
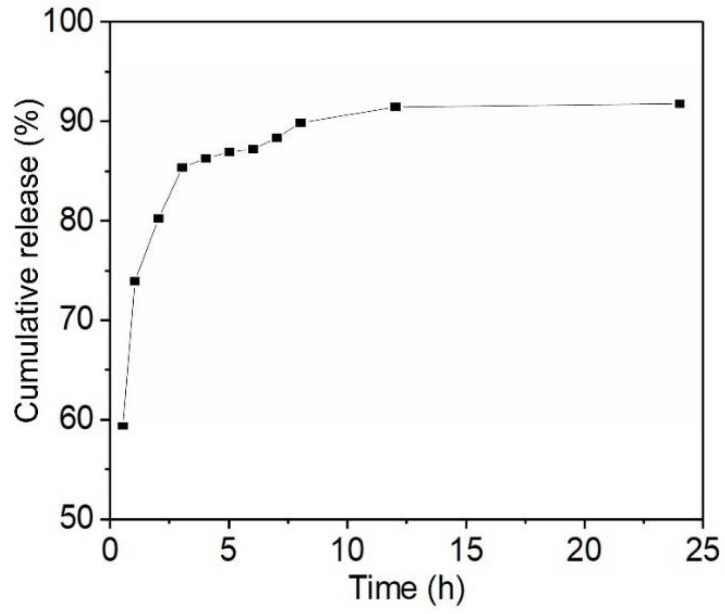
Cumulative drug release curve of NaSA-load cellulose-PEI at different time.

To investigate the drug release behaviors, cellulose-PEI was first loaded with drug in a NaSA solution (100 mg/L) at pH of 3 for 24 h to reach the adsorption equilibrium. Then, the time dependent drug release from the NaSA-loaded cellulose-PEI was monitored at 37 °C in the medium of SBF, which had similar compositions to human blood plasma. Unlike those conventional cellulose materials which showed abrupt drug release as soon as in contact with an aqueous medium [30], cellulose-PEI exhibited a remarkable sustained drug release profile. As shown in Figure 7, the drug release of cellulose-PEI increased rapidly in the first 1 h. This should be attributed to the decreased concentration of −NH_3_^+^ as the pH changed from 3 to 7.4, leading to the desorption of NaSA from cellulose-PEI to SBF. Besides, the freeze-dried cellulose-PEI also contained some physically attached NaSA (from the residue drug solution, while deposited on cellulose-PEI after freeze-drying), which could be more easily released to SBF. During 1–4 h, the drug release rate slightly slowed down. It is believed that the physically attached NaSA had been completely released prior to this stage and the released drug should be from PEI-bound NaSA. As the contact time in SBF further increased to 12 h, the drug release rate significantly decreased and the cumulative drug release tended to be stabilized, suggesting that the competition between drug adsorption and release had reached the equilibrium. Impressively, cellulose-PEI displayed a prolonged drug release time of 12 h, which outperformed many other drug carriers derived from cellulose in literatures (Table S1) [30,42,43,44,45].

Moreover, the drug release behaviors of cellulose-PEI were further analyzed with three pharmacokinetic models, including Zero-order (Equation (6)), First-order (Equation (7)) and Higuchi (Equation (8)) [42,46].

(6) $$Q_{t}=\mathrm{kt},$$

(7) $$\frac{M_{t}}{M_{\infty }}=1-\exp\left(-\mathrm{kt}\right),$$

(8) $$Q_{t}={\mathrm{kt}}^{0.5},$$

where M_t_ and M_∞_ are the absolute cumulative amounts of drug released at time t and at infinite time; k, Q_t_, n and t are the kinetics constant, cumulative release ratio, diffusion exponent and release time, respectively.

The fitting parameters were shown in Table 2. It was obvious that the drug release profile of cellulose-PEI fitted well with the First-order model with a correlation coefficient R^2^ of 0.9656, much higher than those of Zero-order model and Higuchi model. The First-order model usually describes the water-soluble drug dissolution in porous matrices, which was well in line with the structure of cellulose-PEI [46].

## 4. Conclusions

In this work, a novel wound dressing material with high drug loading and sustained release properties was developed through electrospinning of cellulose followed by surface modification with PEI. Morphological analysis revealed that the neat electrospun cellulose nonwoven was highly porous, and this structure could be well retained after PEI modification. FT-IR and XPS survey indicated that PEI had been successfully grafted onto the cellulose nanofibers, providing a large number of amino groups on cellulose-PEI for significant improvement of NaSA loading. When immersed in the NaSA solution with a concentration of 100 mg/L, the maximum drug loading capacity of cellulose-PEI could reach 78 mg/g at pH 3, which was 20 times higher than that of neat electrospun cellulose. In addition, the drug adsorption behavior could be well described by the Freundlich isotherm, suggesting that it was a multilayer adsorption process. Moreover, the NaSA-loaded cellulose-PEI membrane could continuously and effectively release the drug in SBF for 12 h, indicative of the remarkable sustained drug release capability. The above results consistently manifested that cellulose-PEI was an attractive biomaterial. Considering the intrinsic biocompatibility, biodegradability, great mechanical property, flexibility and abundant raw material supplies, the system of cellulose-PEI is highly promising in future wound dressing applications where prolonged drug release is desired.

## Figures and Tables

**Table 1:** Some key parameters for the two adsorption models.

	Langmuir Model	Freundlich Model
q_m (mg/g)_	265.25	--
K_L (L/mg)_	0.015	--
n	--	2.39
K_F (L/mg)_	--	13.47
R^2^	0.8580	0.9681

**Table 2:** Some key parameters for the three drug release models.

	Zero-Order Model	First-Order Model	Higuchi Model
k R^2^	0.1223 0.7233	1.7861 0.9656	0.3635 0.2228

## Data Availability

The data presented in this study are available within this article. Further inquiries may be directed to the authors.

## Supplementary Materials

The following are available online at https://www.mdpi.com/article/10.3390/polym14091748/s1. Figur S1: The standard absorbance-concentration curve of NaSA solutions; Table S1: Comparison of sustained drug release times of cellulose-PEI and other cellulose-based drug carriers.
