# Supplementary material for: Polyethylenimine-Functionalized Nanofiber Nonwovens Electrospun from Cotton Cellulose for Wound Dressing with High Drug Loading and Sustained Release Properties
Source: Polymers (Basel). 2022 Apr 26;14(9):1748. doi: 10.3390/polym14091748 (PMC9105497; doi:10.3390/polym14091748)
Supplement: Supplementary file 1 [file polymers-14-01748-s001.zip › polymers-1669260-supplementary.pdf]

Supplementary Information

Polyethylenimine-Functionalized Nanofiber Nonwovens Electrospun from Cotton Cellulose for Wound Dressing with High Drug Loading and Sustained Release Properties

Qunhao Wang [1], Mei Li [1], Zhuo Zheng [1], Yan Niu [1], Xiaolin Xue [1], Chenghong Ao [1,2], Wei Zhang [1,3,*], Canhui Lu [1,3,*]

1 State Key Laboratory of Polymer Materials Engineering, Polymer Research Institute at Sichuan University, Chengdu 610065, China.; wangqunhao@stu.scu.edu.cn (Q.W.); limei991010@163.com (M.L.); zhuo_zheng@scu.edu.cn (Z.Z.); 17839918692@163.com (Y.N.); xuexiaolin@stu.scu.edu.cn (X.X.); chenghongaocd@163.com (C.A.)

2 Faculty of Environmental Science & Engineering, Kunming University of Science & Technology, Kunming 650500, P. R. China

3 Advanced Polymer Materials Research Center of Sichuan University, Shishi 362700, China;

* Correspondence: weizhang@scu.edu.cn (W.Z.); canhuilu@scu.edu.cn (C.L.); Tel.: +86-28-85460607 (W.Z.); Fax: +86-28-85402465 (W.Z.)

1. Figure

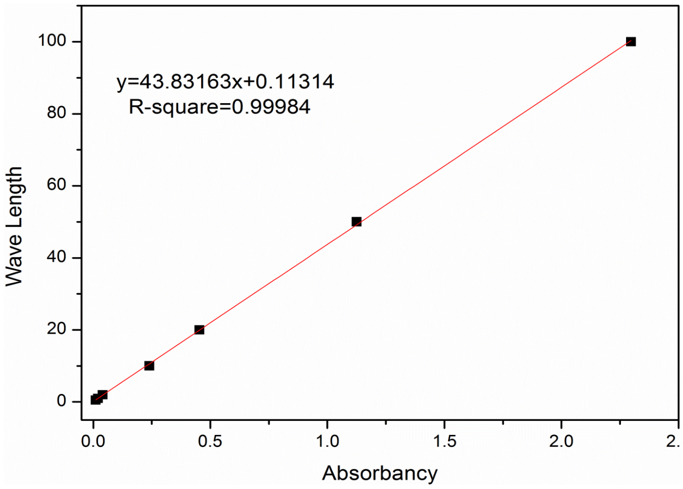

Figure S1. The standard absorbance-concentration curve of NaSA solutions.

2. Table

Table S1. Comparison of sustained drug release times of cellulose-PEI and other cellulose-based drug carriers.

	Drug	Drug release time	Ref
Cellulose nanocrystal/chitosan hydrogel	theophylline	6 h	[1]
Trimethylsilyl cellulose film	diclofenac	4 h	[2]
Cellulose nanofibril aerogel	sodium salicylate	2 h	[3]
Bacterial cellulose/acrylic acid hydrogel	bovine serum albumin	8 h	[4]
Bacterial cellulose	Diclofenac sodium	10 h	[5]
Cellulose-PEI membrane	sodium salicylate	12 h	This work

References

1. Xu, Q.; Ji, Y.; Sun, Q.; Fu, Y.; Xu, Y.; Jin, L. Fabrication of Cellulose Nanocrystal/Chitosan Hydrogel for Controlled Drug Release. Nanomaterials 2019, 9, 253.

2. Maver, T.; Maver, U.; Mostegel, F.; Griesser, T.; Spirk, S.; Smrke, D.M.; Stana-

Kleinschek, K. Cellulose Based Thin Films as a Platform for Drug Release Studies to Mimick Wound Dressing Materials. Cellulose 2015, 22, 749–761.
3. Mohd Amin, M.C.I.; Ahmad, N.; Halib, N.; Ahmad, I. Synthesis and Characterization of Thermo- and PH-Responsive Bacterial Cellulose/Acrylic Acid Hydrogels for Drug Delivery. Carbohydrate Polymers 2012, 88, 465–473.
4. Zhao, J.; Lu, C.; He, X.; Zhang, X.; Zhang, W.; Zhang, X. Polyethylenimine-Grafted Cellulose Nanofibril Aerogels as Versatile Vehicles for Drug Delivery. ACS Appl. Mater. Interfaces 2015, 7, 2607–2615.
5. Adepu, S.; Khandelwal, M. Ex-Situ Modification of Bacterial Cellulose for Immediate and Sustained Drug Release with Insights into Release Mechanism. Carbohydrate Polymers 2020, 249, 116816.
